# Enhanced Magnetic Properties in Antiferromagnetic-Core/Ferrimagnetic-Shell Nanoparticles

**DOI:** 10.1038/srep09609

**Published:** 2015-04-15

**Authors:** Marianna Vasilakaki, Kalliopi N. Trohidou, Josep Nogués

**Affiliations:** 1 Institute of Nanoscience and Nanotechnology, NCSR “Demokritos”, Aghia Paraskevi, Attiki, 15310, Greece; 2 ICN2 - Institut Catala de Nanociencia i Nanotecnologia, Campus UAB, 08193 Bellaterra (Barcelona), Spain; 3 ICREA - Institució Catalana de Recerca i Estudis Avançats, Barcelona, Spain

## Abstract

Bi-magnetic core/shell nanoparticles are gaining increasing interest due to their foreseen applications. Inverse antiferromagnetic(AFM)/ferrimagnetic(FiM) core/shell nanoparticles are particularly appealing since they may overcome some of the limitations of conventional FiM/AFM systems. However, virtually no simulations exist on this type of morphology. Here we present systematic Metropolis Monte Carlo simulations of the exchange bias properties of such nanoparticles. The coercivity, H_C_, and loop shift, H_ex_, present a non-monotonic dependence with the core diameter and the shell thickness, in excellent agreement with the available experimental data. Additionally, we demonstrate novel unconventional behavior in FiM/AFM particles. Namely, while H_C_ and H_ex_ decrease upon increasing FiM thickness for small AFM cores (as expected), they show the opposite trend for large cores. This presents a counterintuitive FiM size dependence for large AFM cores that is attributed to the competition between core and shell contributions, which expands over a wider range of core diameters leading to non-vanishing H_ex_ even for very large cores. Moreover, the results also hint different possible ways to enhance the experimental performance of inverse core/shell nanoparticles for diverse applications.

In recent years, the development of novel synthetic routes, allowing the delicate control of the morphology of nanoparticles, has triggered the interest in more complex magnetic structures such as bi-magnetic core/shell nanoparticles^1^. Among them, ferromagnetic (FM)/antiferromagnetic (AFM) systems are the most studied due to their exchange bias properties (e.g., loops shifts or coercivity enhancement)^2^. These systems not only combine the properties of the different constituents, but the core/shell interactions can provide an additional degree of freedom to improve the properties, thus opening new avenues for innovative applications of magnetic nanoparticles, ranging from energy storage^3^,^4^,^5^,^6^,^7^,^8^ to biomedicine^9^,^10^,^11^. Interestingly, currently, there is an increasing interest in, so-called, inverted structures (see Fig. 1), where the shell is FM or ferrimagnetic (FiM) and the core is AFM, containing for example Mn oxides^12^,^13^,^14^,^15^,^16^,^17^,^18^, Fe oxides^19^,^20^,^21^,^22^,^23^,^24^,^25^,^26^,^27^,^28^,^29^, Co oxides^30^,^31^,^32^,^33^,^34^,^35^,^36^, Cr oxides^37^,^38^,^39^, metallic FePt^40^ or even multiferroic BiFeO_3_ (Refs. 41,42). This type of inverted structures overcomes some of the limitations of conventional systems, since the AFM structure (and thus its magnetic properties) can be much better controlled in the core than in the shell (where usually it is forced to grow in non-ideal conditions). It has been demonstrated, experimentally and theoretically, that the poor crystallinity of the AFM counterpart can result in considerably inferior exchange bias properties^43^,^44^. In fact, inverted structures have already demonstrated very large coercivities and loop shifts, tunable blocking temperatures, enhanced Néel temperatures or proximity effects^12^,^13^,^14^,^15^,^16^,^17^,^18^,^19^,^20^,^21^,^22^,^23^,^24^,^25^,^26^,^27^,^28^,^29^,^30^,^31^,^32^,^33^,^34^,^35^,^36^,^37^,^38^,^39^,^40^,^41^,^42^ and have been proposed as potential magnetoelectric random access memories^41^. However, despite their potential, systematic studies of size effects (i.e., core diameter or shell thickness) are still rather scarce^12^,^16^,^22^,^25^,^33^,^34^,^35^. Remarkably, similar effects of the role of the position of the different magnetic phases (core vs shell) also arise in other types of bi-magnetic core/shell nanoparticles such as hard-FM/soft-FM vs. soft-FM/hard-FM nanoparticles, where systems with the hard counterpart in the core can have enhanced or different properties with respect to the ones with soft-FM cores^45^,^46^. Thus, understanding the role of the position of the diverse magnetic phases (core vs. shell) is of chief importance in the development of novel applications of bi-magnetic core/shell nanoparticles.

From the magnetic point of view, one can identify two main types of inverted structures depending on the transition temperature of the materials. Thus, “single inverted” systems are those where the Curie temperature of the FM, T_C_, is larger than the Néel temperature of the AFM, T_N_, i.e., T_C_ > T_N_, e.g., FeO/Fe_3_O_4_. On the other hand, if T_N_ > T_C_, the systems are usually denoted “doubly inverted”, e.g., MnO/Mn_3_O_4_. Although this type of structure is seldom studied in thin film systems, the available results evidence rather interesting properties^47^,^48^. Similarly, doubly inverted core/shell nanoparticles exhibit some novel properties such as a non-monotonic dependence of the coercivity and the loop shift on the core size^12^.

From the numerical simulation point of view, the mechanisms and the factors that influence the exchange bias behavior in conventional core/shell nanoparticles, i.e., soft FM core/hard AFM^49^,^50^ or FiM shell nanoparticles^46^,^51^,^52^ have been fairly investigated^53^,^54^. These studies support that the exchange bias field depends mainly on the structure of the interface (uncompensated spins) and the coercive field on the particle size. Studies of inverted, AFM/FM, structures (hard AFM/soft FM) are far scarcer^55^,^56^ and, in fact, there are no reports on doubly inverted structures. Notably, the factors responsible for the observed enhanced magnetic behavior of the inverse structure are largely unknown.

Herein, we investigate the core and shell size dependence of the magnetic properties of hard FiM nanoparticles in doubly inverted (T_N_ > T_C_) AFM/FiM core/shell structures using Monte Carlo (MC) simulations. We consider a broad range of core sizes and shell thicknesses to elucidate the optimum conditions for improved properties for diverse possible applications. The results clearly replicate the non-monotonic dependence of the exchange bias properties observed experimentally. Namely, for very small core sizes both coercive (H_C_) and exchange bias (H_ex_) sharply increase as the core size becomes larger. However above a certain size H_C_ and H_ex_ start to decrease, thus exhibiting a maximum. Further, they show that the dependence of the magnetic properties on the FiM thickness depends critically on the size of the AFM core, leading to H_C_ and H_ex_ proportional to the FiM thickness for large AFM cores. The origin of these unusual effects is shown to arise from the competition between the AFM core spins and the hard FiM shell.

## Results

**Figure 1:**
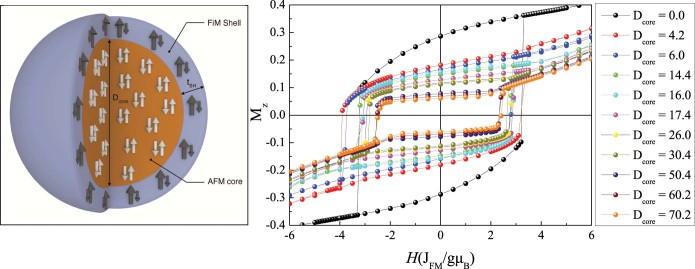
Schematic representation and core size dependence of the hysteresis loops. (left) Schematic representation of the inverse AFM/FiM core/shell structure and (right) hysteresis loops of the doubly inverted nanoparticles, for different AFM core sizes (D_core_) and constant FiM shell thickness of four lattice spacings.

**Figure 2:**
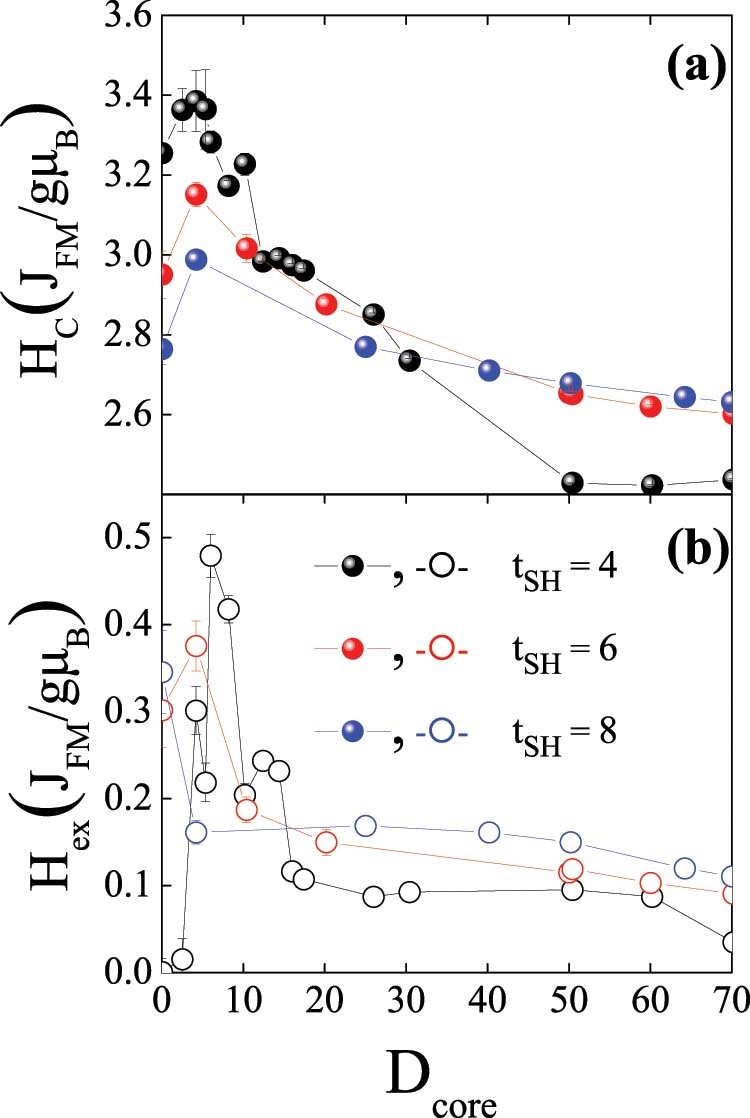
Core size dependence of the coercivity and the exchange bias. Dependence of the coercivity, H_C_ (a) and exchange bias shift, H_ex_ (b) on the AFM core size, D_core_ for the doubly inverted (T_C_ < T_N_ – J_core_ > J_shell_) structures for t_SH_ = 4, 6 and 8.

Shown in the right panel of Fig. 1 are the hysteresis loops for doubly inverted nanoparticles for various core diameters, D_core_, and a constant shell thickness, t_SH_ of four lattice spacings, t_SH_ = 4. The saturation magnetization of the loops decreases for larger D_core_ as expected from the zero net magnetic moment of the AFM core. Moreover, it can be seen that the loops exhibit both H_C_ and H_ex_, which have different behavior for the diverse D_core_. The dependence of H_C_ and H_ex_ on D_core_ for t_SH_ = 4, 6, 8 is shown in Fig. 2.

**Figure 3:**
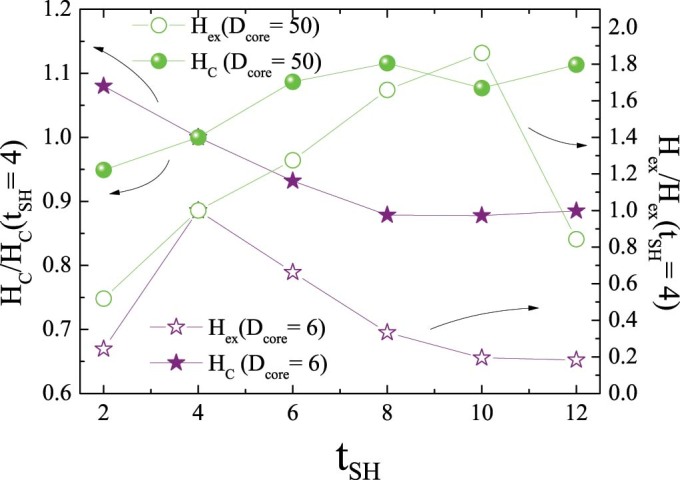
Shell size dependence of the coercivity and the exchange bias. Dependence of the normalized H_C_ and H_ex_ on t_SH_ for D_core_ = 6 and 50.

The results clearly show that inverted structures can result in sizable loop shifts and coercivity enhancements, similar to conventional FM/AFM structures^2^. Nevertheless, contrary to conventional systems, a strong non-monotonic behavior is observed. Interestingly, both H_C_ and H_ex_ exhibit maximum values for rather small D_core_. Moreover, the maximum H_C_ is obtained for very small D_core_ (e.g., D_core_ = 4.2 for t_SH_ = 4) whereas the maximum H_ex_ is observed for slightly larger D_core_ (e.g., D_core_ = 6 for t_SH_ = 4). These results are in qualitative agreement with the experimental doubly inverse MnO/Mn_3_O_4_ nanoparticles case, which also shows an analogous non-monotonic dependence of H_C_ and H_ex_ on D_core_ (Ref. 12). Remarkably, both H_C_ and H_ex_ decrease considerably slow with increasing D_core_. In fact, H_C_ and H_ex_ stabilize for large D_core_ values and H_ex_ does not vanish even for D_core_ = 70. The overall behavior of both H_C_ and H_ex_ is similar for the three t_SH_. Explicitly, they all exhibit non-monotonic behavior with maxima both in H_C_ and H_ex_ at small D_core_ values. However, a more careful analysis of the data reveals several appealing features. Concerning H_C_, it decreases slower as D_core_ increases for thicker t_SH_, leading to a crossover behavior for large D_core_. Interestingly, although for small D_core_ H_C_ decreases for thicker t_SH_, the coercivity enhancement, i.e., ΔH_C_/H_C_, becomes larger for thicker shells, reaching almost 10% for t_SH_ = 8. Regarding H_ex_, two remarkable features are worth mentioning. First, the maximum H_ex_ is obtained for smaller D_core_ as t_SH_ increases. Secondly, similar to H_C_, H_ex_ also exhibits a cross over behavior. As expected from the inverse FM thickness dependence of exchange bias observed in thin films or conventional core/shell particle^2^, for small D_core_ H_ex_ decreases for larger t_SH_. However, this trend is reversed for large D_core_ and, counterintuitively, H_ex_ increases for larger t_SH_. This is summarized in Fig. 3, where it can be clearly seen that while for small core sizes H_C_ and H_ex_ exhibit the conventional inversely proportional to t_SH_ behavior (i.e., ~∝1/t_SH_), for large cores the behavior is against what is expected for an interface effect and is proportional to t_SH_ (i.e., ~∝ t_SH_). This trend is somewhat broken for H_ex_ for exceedingly small t_SH_ in the case of small D_core_ and for large t_SH_ in the case of large D_core_.

The simulations have revealed a number of notable results which, in some cases, are in clear contrast with conventional exchange bias wisdom in thin film and conventional FM/AFM core/shell nanoparticles: (i) both H_C_ and H_ex_ exhibit a strong non-monotonic behavior with D_core_; (ii) the maximum H_C_ and H_ex_ is obtained for rather small D_core_, with sizes comparable to those of the shell; (iii) the largest H_C_ is obtained for smaller D_core_ than for H_ex_; (iv) although H_ex_ and H_C_ increase for thinner t_SH_ for small D_core_, they show an opposite behavior at large D_core_.

## Discussion

**Figure 4:**
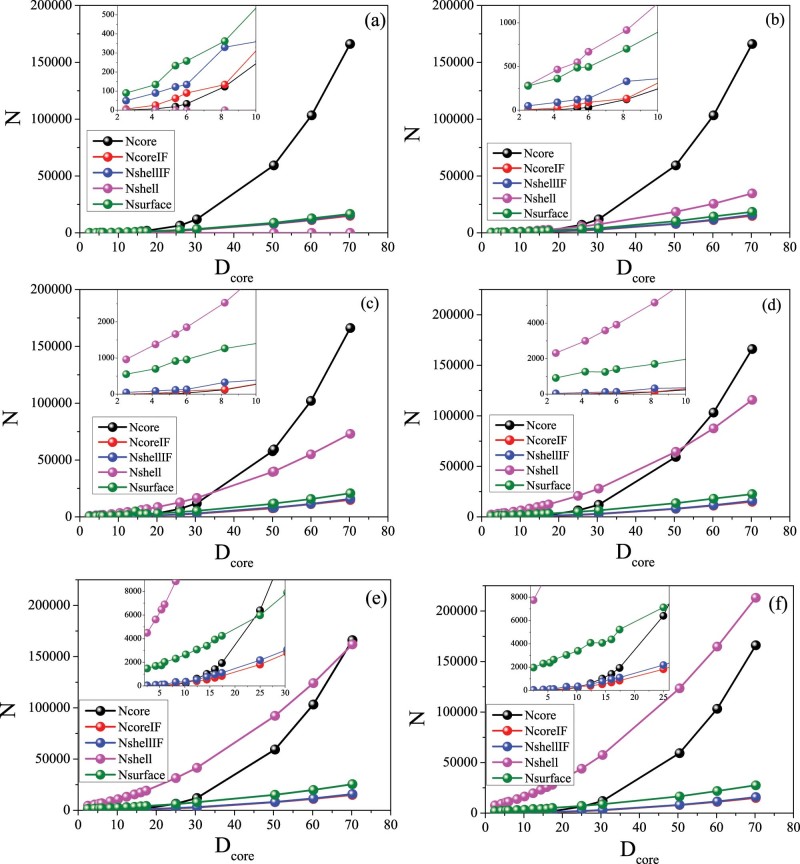
Size dependence of the total number of spins. Number of spins (N) in each region for various core diameters and shell thickness 2 (a), 4 (b), 6(c), 8(d), 10(e) and 12(f) lattice spacings. Shown in the insets are enlarged views of N vs. D_core_ for low D_core_.

**Figure 5:**
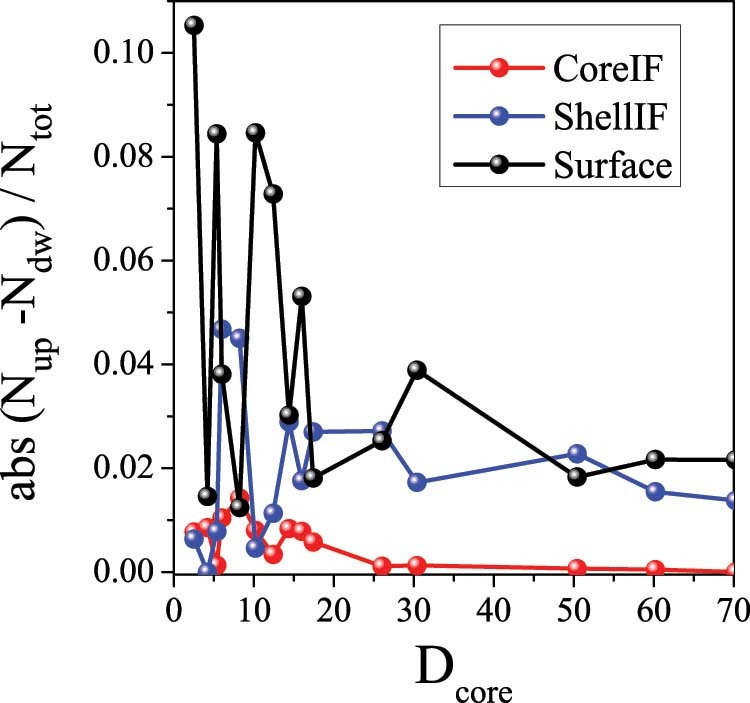
Core size dependence of the number of uncompensated spins. Absolute number of uncompensated spins of the core IF, shell IF and surface normalized to the total number of spins, as a function of the core diameter, D_core_ (for t_SH_ = 4).

To unveil the origin of these novel effects, the number of total and uncompensated spins in the core, interface, shell and surface have been evaluated. Note that uncompensated spins are those spins that due to their local environment of reduced number of neighbors feel a smaller mean field and can thus act more independently. As can be seen in the Fig. 4, the relative number of spins in the different positions with respect to the total number of spins depends strongly on the D_core_ and t_SH_ dimensions. Plotted in Fig. 5 is the absolute number of uncompensated spins N_up_-N_dw_ as a function of the core size, for t_SH_ = 4, normalized to the total number of spins, N_tot_, for the core interface (IF), the shell IF and the surface, taking into account the fact that at the surface and the shell IF the spin magnitude is 1 and 1.5 respectively in the two sublattices. Hence, for the core IF region the normalized number of uncompensated spins is Abs[(N_AFMup_-N_AFMdw_)/(N_AFMup_ + N_AFMdw_ + N_FiMup_ + N_FiMdw_*1.5)] while in the other regions is Abs[(N_FiMup_-N_FiMdw_*1.5)/(N_AFMup_ + N_AFMdw_ + N_FiMup_ + N_FiMdw_*1.5)]. Notably, the notation up and down spins, introduced firstly by Néel^57^, is just schematic to indicate the spins in the two sublattices. Consequently, in the simulations, where the anisotropy parameters are not particularly strong anywhere in the nanoparticle, these spins do not actually act collinearly. Moreover, it should be emphasized that the number of spins and uncompensated spins is solely governed by geometry. Thus, it is important to emphasize that observed fluctuations in the number of uncompensated spins, especially for small core sizes are due to geometrical effects.

For very small core sizes, in the range between 2.5–5.35 lattice spacings, the number of uncompensated spins at the core IF and shell IF is very small, while the number of uncompensated spins at the surface of the shell is considerably larger (Fig. 5). Thus, for very small D_core_ H_ex_ is dominated by the uncompensated spins from the surface of the FiM shell. Although the number of surface spins is relatively small their radial character keeps them pinned on the surface, resulting in finite exchange bias. As D_core_ increases, in the range between 5.35 and 12.4 lattice spacings, the number of uncompensated spins in both the core IF and the shell IF increases (Fig. 5). The maximum of the H_ex_ corresponds to the core size that gives the maximum value of the number of uncompensated spins at the shell interface and a sizable contribution of the core IF and surface uncompensated spins. Thus, the shell IF and the surface mainly contribute to the maximum H_ex_. As D_core_ becomes even larger, for core sizes in the range 12.4–25 lattice spacings, the number of uncompensated spins of the core IF becomes almost negligible and finally vanishes for D_core_ >25 lattice spacings, while the average number of uncompensated spins at the shell IF and the surface slowly decreases, leveling off for very large D_core_. This behavior is similar to one observed for H_ex_ (Fig. 2). Consequently, for large D_core_ H_ex_ is controlled mainly by the shell. Thus, the evolution of the uncompensated spins can qualitatively explain the overall dependence of H_ex_ on D_core_. Interestingly, the large exchange J_core_ of the doubly inverted structure results in an extra anisotropy in the core spins. This gives rise to a stronger resistance of the core spins to be dragged by the reversal of the shell spins, leading to a non-vanishing H_ex_ even for large D_core_ (in the range 30–60.2 lattice spacings). For this D_core_ range, although the number of core spins dominates, the shell spins still contribute to the exchange bias properties, acting as a pining center and therefore competing with the core spins over a wide range of core sizes, where the shell still has a sizeable contribution.

These arguments can also account for the non-monotonic behavior of H_ex_(t_SH_) for the different D_cores_ observed in Fig. 3. Namely, in the case of small D_core_ as t_SH_ becomes exceedingly small (e.g. t_SH_ = 2) the number of surface spins and shell interface spins (see Fig. 4) dominate over core spins, leading to a H_ex_ reduction. On the other hand for large D_cores_, as t_SH_ becomes sufficiently large the competition between core spins and shell spins increases resulting in an increase of H_ex_.

Concerning H_C_, for very small core sizes we have contribution mainly from the shell and the maximum occurs for the core size where the total number of spins from the shell plays the dominant role (see inset in Fig. 5). For larger core sizes, the extra anisotropy induced by the shell acting as pinning center (as N_core_ starts to increase) results in a slow decrease H_C_ as D_core_ is increased.

Regarding the role of the shell thickness, given the larger shells in the t_SH_ = 6 and 8 (compared to t_SH_ = 4) the competition between the core and shell contributions (i.e., when then number of core spins become larger than the number of shell spins; see Fig. 4) occurs at different D_core_. The boundaries are roughly D_core_ ~ 25, 30 and 50 for t_SH_ = 4, 6 and 8, respectively. Consequently, the decrease of H_ex_ and H_C_ for large D_core_ is pushed to larger D_core_ as t_SH_ increases. This gives rise to the crossover from the conventional FM thickness dependence of exchange bias systems (i.e., t_FM_ ↑ → H_ex_, H_C_ ↓) at small D_core_ to the counterintuitive reversed behavior (t_FM_ ↑ →H_ex_, H_C_ ↑) for large D_core_.

**Figure 6:**
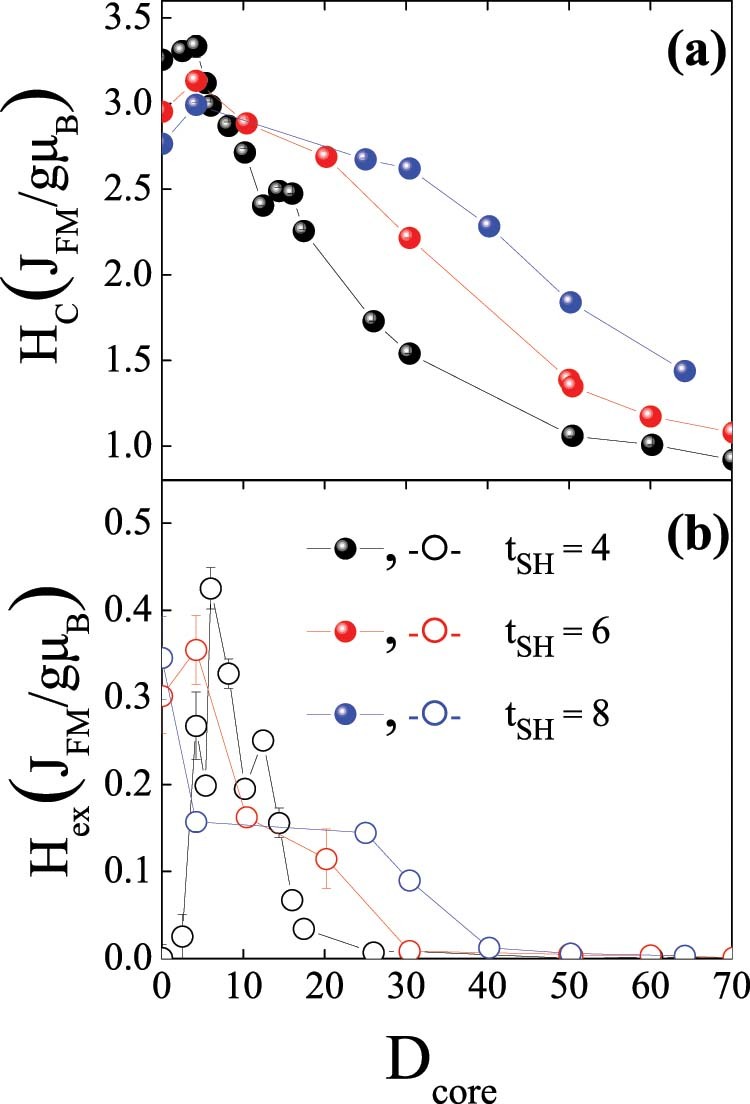
Core size dependence of the coercivity and the exchange bias. Dependence of the coercivity, H_C_ (a) and exchange bias shift, H_ex_ (b) on the AFM core size, D_core_ for the single inverted (T_C_ > T_N_ – J_core_ < J_shell_) structures for t_SH_ = 4, 6 and 8.

Notably, this unusual behavior is to some extent different in single inverted AFM core/FiM shell nanoparticles shown in Fig. 6 (i.e., T_N_ < T_C_ – J_core_ < J_shell_). Namely, while H_C_ exhibits a H_C_ ∝ t_SH_ dependence for large D_cores_ similar to the doubly inverted case, H_ex_ shows this inverse behavior only in a very narrow range of D_cores_ since H_ex_ vanishes at large D_cores_. In this case since J_core_ is weaker there is no competition between the core and the shell. Thus, the shell drags the core spins with the consequent decrease of H_ex_ and H_C_. Consequently, the doubly inverted structures present improved properties compared to the single inverted ones, especially for large D_cores_.

From the applications point of view some of the features unveiled from the simulations are rather attractive. One possible use of these structures could be to utilize the coercivity enhancement for small D_core_ to improve the performance of permanent magnets^58^. Given the higher H_C_ for thin shells, probably thin shells with small cores would be ideal for this type of purposes. Note that despite the loss of saturation magnetization, M_S_ (due to the zero M_S_ of the AFM core), since the core is so small the energy product (i.e., the figure of merit of a permanent magnet) may actually be improved (similar to what has been observed in AFM/FM composites^59^) in these structures. For recording applications^3^,^4^, where, for example, enhancement of the blocking temperature of very small nanoparticles may be pursued, perhaps larger coercivity enhancement, ΔH_C_ (which implies an increased effective anisotropy) may be more appealing. Thus, thicker shells with small cores would be more appropriate. Note that for this type of applications inverse AFM/FM structures may be more suitable than conventional FM/AFM, since the stray field of the nanoparticles (required for easy detection) would be less attenuated. For applications based on H_C_, doubly inverted structures may be more attractive, especially if large cores are needed. Concerning applications requiring exchange bias, e.g., miniaturized magnetotransport devices^6^,^41^, the optimum D_core_/t_SH_ configuration would depend on the exact H_C_/H_ex_ required. For example, for large H_C_ and H_ex_, then small t_SH_ with moderate D_core_ may be best. Nevertheless for cases where moderate H_C_ and H_ex_ are needed, large t_SH_ and D_core_ would be better.

## Conclusion

In conclusion, the behavior of the exchange bias and the coercive field in doubly inverted AFM core/hard FiM shell nanoparticle systems have been shown to depend on the core size in a different way at various core size ranges. For very small core sizes there is contribution on H_ex_ from the surface uncompensated spins. For moderate core sizes the uncompensated spins of the core and the shell interface also contribute to the exchange bias, resulting in a maximum H_ex_ value. For even larger core diameters the exchange in the core, J_core_ and the AFM character of the core determine H_ex_. For large D_core_ the whole shell plays the role of the shell IF, thus the exchange bias effect for these core sizes increases with shell thickness, in contrast to conventional systems. The study of the role of the shell thickness indicates that a sizable shell contribution is needed to ensure enhancement of the exchange bias properties. The improved magnetic properties can satisfy a range of technological demands.

## Methods

For the study of the magnetic behavior of the nanoparticles we use the Monte Carlo simulation technique and the Metropolis algorithm^53^. We consider spherical nanoparticles of diameter D, expressed in lattice spacings, on a simple cubic lattice with inverted structure, consisting of an AFM core and a FiM shell (see Fig. 1-left panel). We take into account explicitly the microstructure of the system in an atomic scale. The spins of the nanoparticles are located at each lattice site of the core, the interface, the shell and the surface. They interact with nearest neighbours through Heisenberg exchange interactions and at each crystal site they experience a uniaxial anisotropy. At the surface of the particles, the crystal symmetry is reduced and consequently the anisotropy is stronger than in the bulk^60^,^61^. The FiM shell is considered as a layer surrounding the core.

In the presence of an external magnetic field, the total energy of the system is
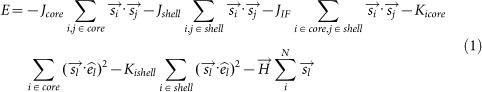
Here 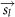 is the atomic spin at site i and 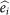 is the unit vector in the direction of the easy axis at site i. We consider the magnitude of the atomic spins in the two AFM sublattices equal to 1 and in the two FiM sublattices of the shell to be equal to 1 and 1.5, respectively. The first term in Eq. 1 gives the exchange interaction between the spins in the AFM core, while the second term gives the exchange interaction between the spins in the FiM shell. We consider two different cases, (i) single inverted with T_C_(shell) > T_N_(core) and (ii) doubly inverted with T_N_(core) > T_C_(shell). To take into account the difference in transition temperatures, for the first case we consider the exchange coupling constant of the core as J_core_ = −0.5 J_FM_ and that of the shell as J_shell_ = −1.5 J_FM_ where J_FM_ is the exchange coupling constant of a pure ferromagnet J_FM_ = 1 as reference value. In the doubly inverted structures, J_shell_ is maintained the same, but J_core_ in increased to account for the larger T_N_. Thus, the exchange coupling constants are set to J_core_ = −3.0 J_FM_ and J_shell_ = −1.5 J_FM_. The third term gives the exchange interaction at the interface between the core and the shell. The interface includes the last layer of the AFM core and the first layer of the FiM shell. The exchange coupling constant of the interface J_IF_ is taken equal to that of the shell J_SH_. The forth term gives the anisotropy energy of the AFM core, K_C_. If the site i lies in the outer layer of the AFM core K_icore_ = K_IF_ and K_icore_ = K_C_ elsewhere (with K_C_ = 0.05 J_FM_ -i.e., a soft AFM- and K_IF_ = 0.5 J_FM_). The fifth term gives the anisotropy energy of the FiM shell, which is taken as K_SH_ = 0.5 J_FM_. If i lies in the outer layer (i.e., the surface) of the shell then the anisotropy is taken as K_S_ = 1.4 J_FM_, which is assumed to be radial (rather than uniaxial). The last term in Eq. 1 is the Zeeman energy.

The aim of our model is to qualitatively reproduce the magnetic behavior of this new class of advanced nanomaterials, therefore the parameters used in the simulations for the exchange and the anisotropy constants were chosen from a careful analysis of the experimental magnetic behavior of the nanoparticle system MnO (AFM)/Mn_3_O_4_ (FiM) (i.e., the most studied doubly-inverse system)^12^. Note that since ab initio electronic calculations cannot be performed for this type of complex nanoparticles the parameters were estimated by modifying the bulk values Mn_3_O_4_ and MnO taking into account the lower crystal symmetry at the interface and at the surface, which results in higher effective anisotropy energy in these regions. Nevertheless, the K parameters are weighted by their corresponding volume^49^. The exchange coupling parameters for the core, the interface, the shell and the surface are considered here to be the same for all the different core and shell sizes since the exchange coupling parameters are short ranged and hence the influence of the second or third neighbors is almost negligible^62^. Moreover, it is worth noting that experimental studies of the detailed chemical characterization of MnO/Mn_3_O_4_ core/shell nanoparticles indicate that the interface between MnO and Mn_3_O_4_ is rather sharp^63^, thus justifying our model.

Importantly, the chosen parameters roughly maintain the main experimental characteristics (i.e., T_C_(Mn_3_O_4_)/T_N_(MnO) ~ 1/3 and K_FiM_(Mn_3_O_4_)/K_AFM_(MnO) ~ 50) with simulated values of T_C_(Mn_3_O_4_)/T_N_(MnO) ~ 1/3 and K_FiM_(Mn_3_O_4_)/K_AFM_(MnO) ~ 10. Note that the smaller than bulk ratio K_FiM_(Mn_3_O_4_)/K_AFM_(MnO) used in the simulations was chosen to take into account the possible worsening of the magnetic properties of the shell material due to structural deterioration of shell. Note, for example, that although the Mn_3_O_4_ shell in MnO/Mn_3_O_4_ core/shell nanoparticles exhibits a T_C_ close to bulk values, their H_C_ is usually smaller (i.e., lower K(Mn_3_O_4_))^12^,^16^,^17^,^18^.

**Figure 7:**
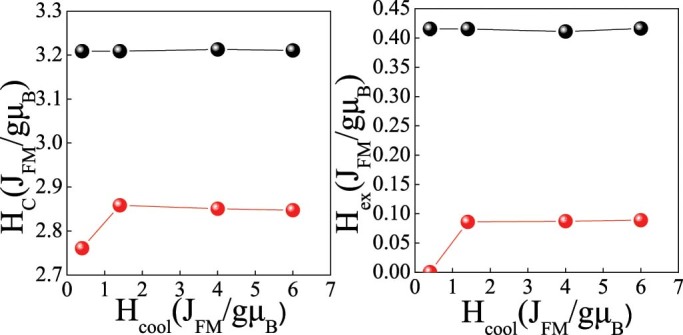
Effect of the cooling field. Dependence of coercivity, H_C_ (left) and exchange bias, H_ex_ (right) on the cooling field, H_cool_, for D_core_ = 8.2 (squares) and 26 (circles), with t_SH_ = 4.

We perform our simulations of the hysteresis loops on isolated nanoparticles using the Monte Carlo (MC) simulation technique with the implementation of the Metropolis algorithm^64^. The hysteresis loops are calculated after a field cooling procedure starting at temperature T = 7.0 J_FM_/k_B_ down to T_f_ = 0.01 J_FM_/k_B_, at a constant rate under a static magnetic field H_cool_ = 4.0 J_FM_/gμ_B_ directed along the z-axis. The value of H_cool_ was selected as the optimum value to observe maximum H_ex_ and H_C_ as Fig. 7 shows. The hysteresis loop shift on the field axis gives the exchange field H_ex_ = -(H_right_+H_left_)/2. The coercive field is defined as H_C_ = (H_right_- H_left_)/2. H_right_ and H_left_ are the points where the loop intersects the field axis. The coercive H_C_ and exchange H_ex_ fields are given in dimensionless J_FM_/gμ_B_ units and the temperature T in J_FM_/k_B_.

In the Monte Carlo method, at each Monte Carlo step we select, at random, an atomic spin from the N spins of the nanoparticle and we make a small change in its orientation. This attempted change is accepted if it leads to the lowering of the system's energy or with a certain probability that corresponds to the Boltzmann probability. The process is repeated until equilibrium is reached. We have used 10^4^ MC steps per spin (MCSS) at each field step and the results were averaged over 50–200 different samples (namely random numbers) depending on the size of the nanoparticle and the fluctuations in the values of the calculated fields. The standard deviation of the averages is depicted in the plots as error bars.

## Author Contributions

K.N.T. and J.N. designed the experiments. M.V. and K.N.T. performed the simulations. K.N.T. and J.N. wrote the manuscript. All authors contributed to the analysis and discussion.
